# The *reg4* Gene, Amplified in the Early Stages of Pancreatic Cancer Development, Is a Promising Therapeutic Target

**DOI:** 10.1371/journal.pone.0007495

**Published:** 2009-10-16

**Authors:** Aude Legoffic, Ezequiel Calvo, Carla Cano, Emma Folch-Puy, Marc Barthet, Jean Robert Delpero, Montse Ferrés-Masó, Jean Charles Dagorn, Daniel Closa, Juan Iovanna

**Affiliations:** 1 INSERM U.624, Stress Cellulaire, Parc Scientifique et Technologique de Luminy, Marseille France; 2 Molecular Endocrinology and Oncology Research Center, CHUL Research Center, Québec, Canada; 3 Experimental Pathology Department, IIBB-CSIC-IDIBAPS, CIBERehd, Barcelona, Spain; 4 Département de Chirurgie Oncologique, Institut Paoli-Calmettes, Marseille, France; Louisiana State University, United States of America

## Abstract

**Background:**

The aim of our work was to identify the genes specifically altered in pancreatic adenocarcinoma and especially those that are altered early in cancer development.

**Methodology/Principal Findings:**

Gene copy number was systematically assessed with an ultra-high resolution CGH oligonucleotide microarray in DNA from samples of pancreatic cancer. Several new cancer-associated variations were observed. In this work we focused on one of them, involving the *reg4* gene. Gene copy number gain of the *reg4* gene was confirmed by qPCR in 14 cancer samples. It was also found with increased copy number in most PanIN3 samples. The relationship betweena gain in *reg4* gene copy number and cancer development was investigated on the human pancreatic cancer cell line Mia-PaCa2 xenografted under the skin of nude mice. When cells were transfected with a vector allowing reg4 expression, they generated tumors almost twice larger in size. In addition, these tumors were more resistant to gemcitabine treatment than control tumors. Interestingly, weekly intraperitoneal administration of a monoclonal antibody to reg4 halved the size of tumors generated by Mia-PaCa2 cells, suggesting that the antibody interfered with a paracrine/autocrine mechanism involving reg4 and stimulating cancer progression. The addition of gemcitabine resulted in further reduction, tumors becoming 5 times smaller than control. Exposure to reg4 antibody resulted in a significant decrease in intra-tumor levels of pAkt, Bcl-xL, Bcl-2, survivin and cyclin D1.

**Conclusions/Significance:**

It was concluded that adjuvant therapies targeting reg4 could improve the standard treatment of pancreatic cancer with gemcitabine.

## Introduction

Pancreatic cancer represents 2% of all new cases of cancer but leads to 5% of all cancer deaths, with a five year survival rate of only 4% [1]. The diagnosis is delayed because of the absence of symptoms and lack of specific markers allowing detection at a potentially curative stage. In the general population pancreatic cancer carries a lifetime risk of approximately 0.5–1% [2]. Genetic predisposition is involved because the lifetime risk of pancreatic cancer is 4.7% for first-degree relatives of pancreatic cancer cases and the risk of pancreatic cancer increases with each affected family member. In addition, it can be inherited as part of a multi-cancer syndrome but the vast majority of pancreatic cancers are sporadic. Finally, tobacco smoking and diseases such as diabetes and especially chronic pancreatitis predispose to pancreatic cancer and about 10% of patients with intrapapillary mucinous neoplasms (IPMNs) will develop pancreatic adenocarcinoma.

Genomic alterations can induce over- or under-expression of genes, with important consequences when oncogenes or tumor suppressor genes are involved. The genetic abnormalities that occur in the precursor lesions [3] as well as during initiation and progression of pancreatic cancer have been partially described [4]–[7]. Indeed, comparative genomic hybridization (CGH) analyses identified frequent gains on chromosomes 1q, 3, 5, 7p, 8q, 11q, 12p, 17q, 19q and 20q, and losses on chromosomes 3p, 6, 8p, 9p, 10q, 13q, 15q, 17p and 18q [8]–[13]. The aim of our work was to identify, using an ultra-high resolution CGH oligonucleotide microarray, the genes specifically altered in pancreatic adenocarcinoma cells and especially those that are altered early in cancer development. Several candidates were identified using this approach, among which the *reg4* gene [14] showed gene copy number gain in 14/14 analyzed samples. This is to our knowledge the first report of *reg4* gene copy number gain in pancreatic cancer.

In this paper we report that the *reg4* gene, positioned on chromosome 1p13.1-p12, is present in increased copy number in pancreatic cancer cells and in late precancerous pancreatic lesions. Studies on a xenografted pancreatic cancer cell line showed that reg4 over-expression stimulates tumor growth and, conversely, that blocking circulating reg4 protein with a specific antibody inhibits tumor growth.

## Results

### The *reg4* gene is present in increased copy number in pancreatic cancer cells and in PanIN 3 precancerous lesions

**Figure 1:**
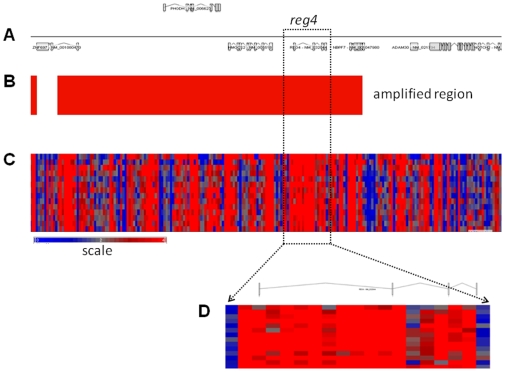
The *reg4* gene is found with increased copy number in pancreatic cancer. Allelic ratios were calculated with the Partek Genomics Suite, version 6.4. HapMap (270 samples) was used to create baseline copy number. Genomic segmentation was utilized to detect copy number gain or loss. Regions were detected using the following segmentation parameters: minimum of 10 genomic markers; segmentation p-value threshold lower than 0.001; a signal to noise equal to 0.3. A. Schematic representation of the *reg4* locus. Positions of the 7 genes present in this locus are indicated: from left to righ: *ZNF697*, *PHGDH*, *HMGCS2*, *REG4*, *NBPF7*, *ADAM30* and *NOTCH2*. B. Position of the copy number gain segment found in all 14 DNA samples from pancreatic cancer, which includes the *reg4* gene. C. Genomic changes in the amplified segment of the *reg4* locus, determined by the Affymetrix Genome-Wide Human SNP Array 6.0 analysis in the 14 pancreatic cancer samples. Genetic gains are shown as red bars and losses as blue bars, grey bars corresponding to 2 copies as indicated in the scale shown underneath. Note the predominance of gains (red) in the *reg4* region. D. Detail of gains/losses in the *reg4* gene and its flanking regions for all 14 analyzed samples.

**Figure 2:**
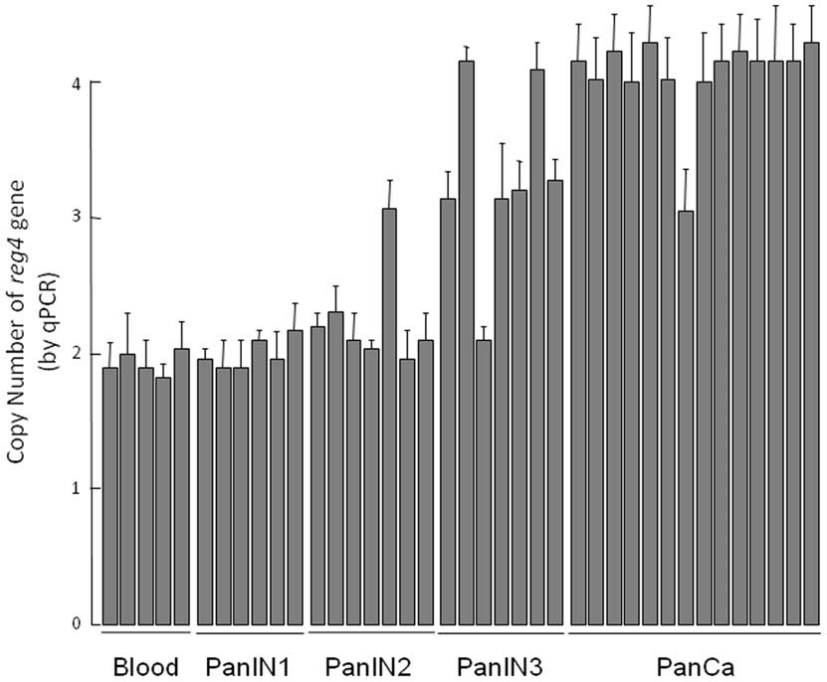
*reg4* gene amplification in pancreatic cancer and precancerous lesions measured by quantitative PCR. Homogeneous populations of cells were carefully microdissected using a Laser Capture Microdissection System. PanINs were graded 1 to 3 as a function of the importance of architectural and cytological abnormalities. DNA from pancreatic cancer samples obtained by endoscopic ultrasound (EUS)-guided fine needle aspiration was used. DNA from blood leucocytes was used as control. *reg4* gene copy number was determined by qPCR performed in triplicate and results were analyzed using the RealQuant software.

Using an Affymetrix microchip-based DNA scanning approach, we identified several genes with an abnormal copy number in DNA from pancreatic cancer cells. We first focused on genes altered in all 14 DNA samples and found that *reg4* showed systematically increased copy number. All microarray data reported in the manuscript is described in accordance with MIAME Guidelines. Complete data describing other DNA abnormalities will be published elsewhere. *reg4* gene increased copy number was particularly interesting because its expression was previously involved in the aggressiveness of several cancers [15]–[17], including pancreas [18]. Figure 1 shows the amplified locus, which comprises the *reg4* gene, in DNA from these patients. We also monitored the copy number of *reg4* in the pancreatic precancerous lesions named PanINs. Combining a laser capture approach and a qPCR method, we measured the number of reg4 copies in the three grades of PanIN lesions and found an increased copy number of *reg4* in 0/6, 1/7 and 6/7 of PanIN1, PanIN2 and PanIN3 lesions, respectively (Figure 2). As control, copy numbers of *TERT* (Telomerase Reverse Transcriptase) and *RPP21* (RNaseP protein p21) genes were assessed on the same DNA samples and two copies were always found (data not shown). Altogether, these data suggest that the *reg4* gene copy number is frequently increased in pancreatic cancer and in the last stage of precancerous lesions (PanIN3) but rarely in earlier stages (PanIN1 and PanIN2).

### reg4 overexpression stimulates cell growth and increases resistance to gemcitabine treatment in MiaPaCa2 cells *in vitro*

**Figure 3:**
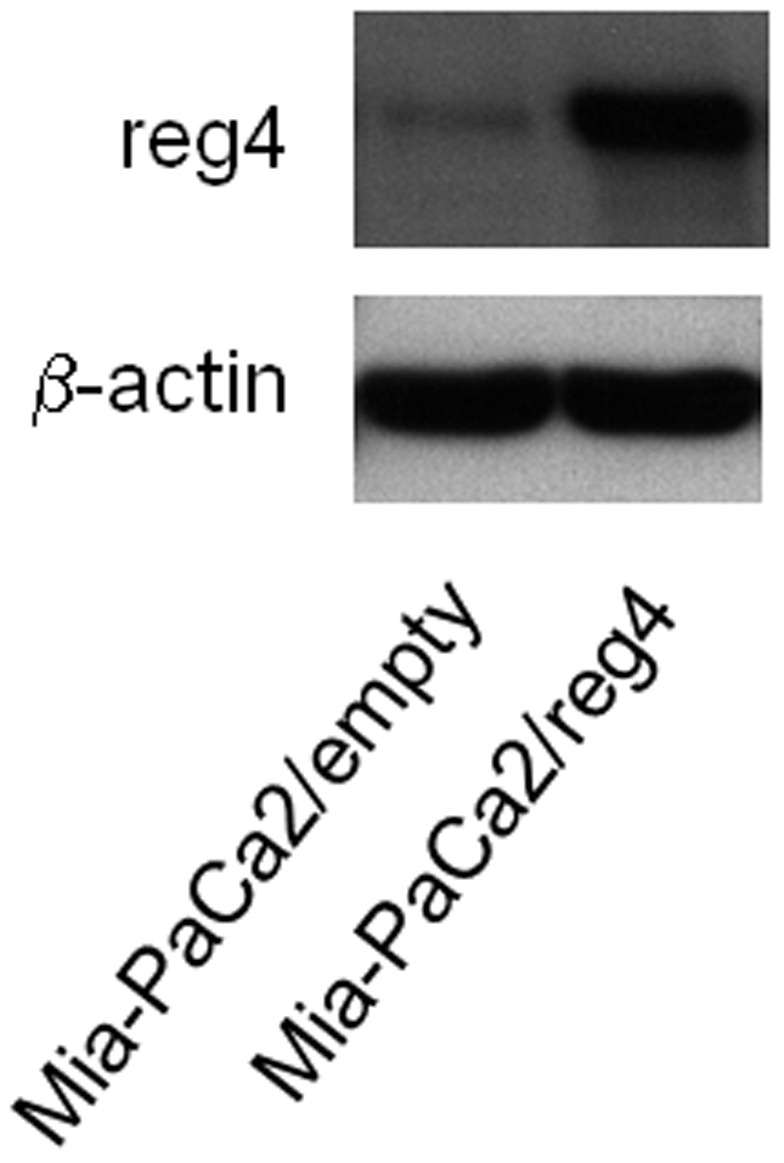
Overexpression of the REG4 protein in Mia-PaCa2 cells. The entire coding sequence of the reg4 cDNA was subcloned into the pLPCX retroviral vector. Phoenix Amphotropic packaging cells were transfected with the retroviral plasmid to obtain the supernatant containing retroviral particles. Target Mia-PaCa2 cells were plated in the presence of the supernatant containing retroviral particles as described in the Material and Methods section. The populations of reg4-expressing Mia-PaCa2 (Mia-PaCa2/reg4) and control cells (Mia-PaCa2/empty), infected with the empty vector, were isolated by puromycin selection. Expression of REG4 was measured by western blot on cell extracts, using the anti-reg4 monoclonal antibody. After development, the membrane was stripped and re-probed for β-actin.

**Figure 4:**
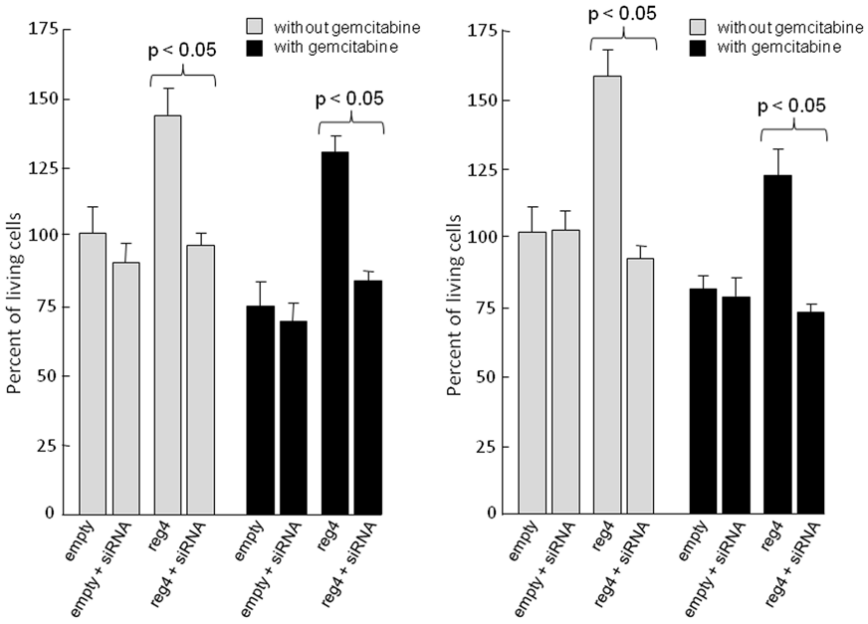
Influence of a reg4 siRNA or a REG4 antibody on growth and response to gemcitabine of Mia-PaCa2 cells overexpressing REG4. Mia-PaCa2/reg4 and Mia-PaCa2/empty cells were transfected with a specific siRNA against reg4 (A) or incubated in the presence of an anti-REG4 monoclonal antibody (B) and their growth and their resistance to gemcitabine treatment was measured by the MTS assay. Results were expressed as percent of untreated Mia-PaCa2/empty cells.

It was previously shown that forced reg4 overexpression *in vitro* increases cell growth rate and resistance to programmed cell death in non-pancreatic cancer-derived cells. We obtained Mia-PaCa2 cells overexpressing reg4 protein (Mia-PaCa2/reg4) by transduction of a recombinant retrovirus (Figure 3) and analyzed their capacity to grow and to resist to the antitumoral drug gemcitabine *in vitro*. We observed that cells expressing reg4 grew about 50% more rapidly than Mia-PaCa2/empty cells. We confirmed that increased cell growth was due to reg4 overexpression by knockingdown reg4 with a specific siRNA transfection and found a loss of this capacity, as shown in Figure 4. Similarly, when Mia-PaCa2 cells were treated with 50 µM gemcitabine for 48 hours, the resistance to the drug was increased in cells overexpressing reg4, compared to control, but lost in cells eventually transfected with a siRNA against reg4 (Figure 4). These results indicate that reg4 is involved in cell growth and resistance to anticancer drugs in pancreas cancer-derived cells as previously suggested in non-pancreatic cells.

REG4 is a 16 kDa secretory protein. To test whether effects of REG4 on cell growth and resistance to gemcitabine treatment were due to its endocrine/paracrine function in pancreatic cells, we added a specific monoclonal antibody against REG4 protein to the culture medium, to block its activity. In these conditions, the effects of reg4 overexpression disappeared almost completely, as shown in Figure 4. These results indicate that REG4 exerts its effects on cell growth and resistance to gemcitabine through an endocrine/paracrine way.

### reg4 overexpression increases tumorigenicity and resistance to gemcitabine treatment

**Figure 5:**
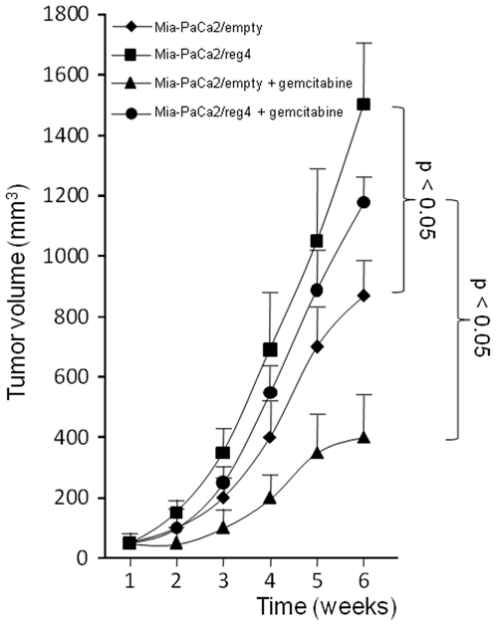
Growth and response to gemcitabine of xenografted Mia-PaCa2 cells overexpressing REG4. Approximately 2×10^6^ Mia-PaCa-2 cells were inoculated subcutaneously with 0.1 ml of Matrigel to nude mice. Gemcitabine (100 mg/kg) was injected intraperitoneally twice a week. Tumor dimensions were measured once weekly and tumor volumes calculated with the formula length × width × depth ×0.5236. Values are expressed as the mean +/− SE of six measurements.

We compared the capacities of Mia-PaCa2/empty and Mia-PaCa2/reg4 cells to form tumors after subcutaneous injection in nude mice. As shown in Figure 5, tumors that formed from cells over-expressing reg4 protein grew more rapidly than cells that do not express the gene. Tumor volume was 70% larger when using Mia-PaCa2/reg4 cells than with control Mia-PaCa2 cells. Interestingly, when mice were treated with gemcitabine, tumors over-expressing reg4 protein displayed increased resistance to the treatment, compared to tumors formed with Mia-PaCa2/empty cells. While the volume of tumors generated with Mia-PaCa2 cells decreases by 60% after gemcitabine treatment, the volume of reg4-expressing cells decreased by 20% only.

### Systemic treatment with an antibody against reg4 decreases tumorigenicity

**Figure 6:**
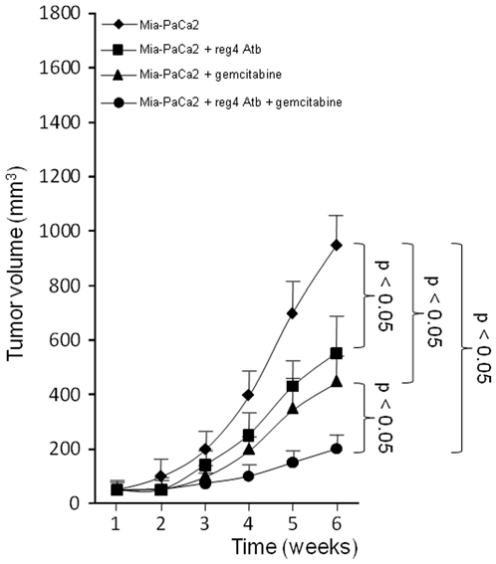
Growth and response to gemcitabine of pancreatic tumors treated with a specific anti-REG4 antibody. Approximately 2×10^6^ Mia-PaCa-2 cells were inoculated subcutaneously with 0.1 ml of Matrigel to nude mice. One day before tumoral cell inoculation, the control group received an intraperitoneal injection of 150 µl of PBS, whereas the assay group received 0.25 mg of reg4 monoclonal antibody in 150 µl of PBS. Vehicle buffer or anti-reg4 antibody was injected weekly in animals. Tumor dimensions were measured once weekly and tumor volumes calculated with the formula length × width × depth ×0.5236. Gemcitabine (100 mg/kg) was injected intraperitoneally twice a week. Values are expressed as mean +/− SE (n = 6).

The next step was to analyze the effect of blocking reg4 with a specific monoclonal antibody on the growth of tumors induced by subcutaneous injection of Mia-PaCa2 cells. As shown in Figure 6, treatment with the reg4 antibody decreased tumor development by about 50%. In addition, combining reg4 antibody treatment with gemcitabine resulted in further reduction of tumor volume. Altogether, these results indicate that targeting reg4 protein might be used in cooperation with gemcitabine to treat pancreatic cancer.

### Intraperitoneal injection of reg4 antibody reduces the levels of antiapototic proteins and cell cycle-associated proteins in Mia-PaCa2-induced tumors

**Figure 7:**
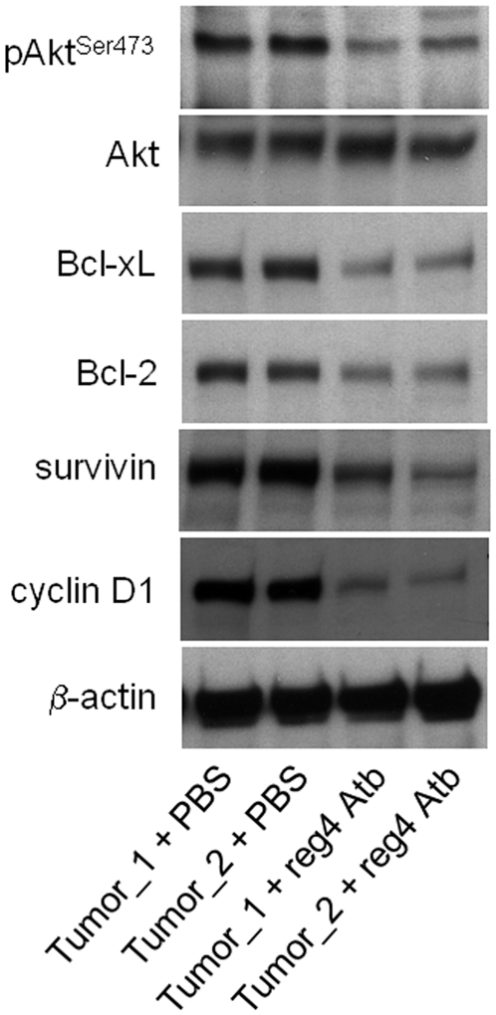
Expression of phosphorylated AKT, Bcl-xL, Bcl-2, surviving and cyclin D1 in pancreatic tumors treated with a specific anti-REG4 antibody. Tumor tissue samples were lysed in a homogenization buffer containing a cocktail of antiproteases. Cell debris and insoluble materials were eliminated by centrifugation and soluble fractions were loaded in Laemmli buffer onto a 12.5% SDS-polyacrylamide gel. The proteins were transferred to nitrocellulose membrane and membrane incubated with the anti phosphorylated AKT (phospho-Ser473), anti-pan AKT, anti-Bcl-2, anti-Bcl-xL, anti-survivin or anti-cyclin D1 antibodies. After development, the membrane was stripped and reprobed for β-actin.

Because systemic treatment with the antibody reduces tumor growth and increases sensitivity to gemcitabine treatment, we used western blot analysis to monitor in tumors the influence of reg4 antibody treatment on the intracellular levels of proteins associated with apoptosis (phosphorylated AKT, Bcl-2, Bcl-xL and survivin) and cell cycle (cyclin D1). As expected levels of phosphorylated AKT, Bcl-2, Bcl-xL and survivin were significantly reduced in tumors from mice treated with the reg4 antibody compared to control. Level of cyclin D1 was also found reduced in reg4 antibody-treated mice (Figure 7). These findings probably account for the observed reduced growth rate.

## Discussion

In this study, our hypothesis was that early genomic abnormalities occurring in pancreatic cancer cells are responsible for the bad prognosis of the patients and for the lack of adequate response to anti-cancer drugs, including gemcitabine. Several DNA regions with altered gene copy number were indeed observed in tumors from patients without detectable metastases at the time of the study. In the present work, we focused our attention on the chromosome region containing the *reg4* gene because it showed increased copy number in all 14 patients analyzed. In addition, an increased copy number of this region was also found in almost all PanIN3, the latest stage of precancerous pancreatic lesions, suggesting that *reg4* gene amplification is an early event in pancreatic cancer development. reg4 is a small secreted protein that contains a single well conserved carbohydrate-recognition domain [14], [15]. reg4 is a member of a multigenic family named *reg* (reviewed in 19). In humans, five structurally-related members have been identified to date and grouped into three subclasses based on the primary structures of the encoded proteins namely REG1A and REG1B (Type 1), REG3A and REG3G (Type 3) and REG4 (Type 4). Contrary to *reg1A*, *reg1B*, *reg3A* and *reg3G* genes, all located on chromosome 2p12, *reg4* is on chromosome 1p13.1-p12. Several reports in the literature suggest a major role for REG4 in cancer. For example, REG4 expression seems to be responsible for cell resistance to anticancer drugs such as 5-fluouracil and methotrexate [15], [20], it promotes AKT phosphorylation and over-expression of the antiapoptotic proteins Bcl-2, Bcl-xL and survivin [21], [22], it activates the EGF receptor/Akt/AP1 signaling pathway [21] and its expression correlates with enhanced peritoneal metastasis in gastric carcinomas [22], [23]. It is systematically overexpressed in cancerous tissues derived from colon [15], [24], stomach [16], prostate [25] and pancreas [18] and in diseases predisposing to colon cancer such as ulcerative colitis [14], [26], Crohn's disease [14] and colorectal adenoma [24]. The early amplification of its gene in pancreatic cancer described in this study (Figures 1 and 2) and its oncogenic activity reported in the literature prompted us to further study the role of REG4 in pancreatic tumorigenicity and in resistance of pancreatic cancer to gemcitabine treatment. The first observation was that pancreatic cancer-derived cells overexpressing REG4 protein grew more rapidly and were more resistant to gemcitabine treatment (Figure 4). The second, more important observation was that, in a xenograft model, tumors induced with Mia-PaCa2 cells expressing reg4 grew more rapidly than control tumors, obtained with native Mia-PaCa2 cells (Figure 5). In addition, we found that the tumors induced by Mia-PaCa2 cells expressing reg4 were more resistant to gemcitabine treatment than control tumors, in agreement with *in vitro* data and previous reports suggesting the involvement of REG4 in cell resistance to anticancer drugs [15], [20] and response to chemoradiotherapy [27]. The third, very exciting result was obtained when mice with Mia-PaCa2-induced tumors were treated with systemic administration of a specific anti-reg4 antibody (Figure 6). We observed an important reduction in tumor size and an increased sensitivity to gemcitabine treatment in mice given once a week anti-reg4 antibody. Because the limited efficiency of pancreatic cancer treatment with gemcitabine might be due to the presence of stroma in tumors [28], we looked whether reg4 overexpression influenced the extent of stroma formation in subcutaneously xenografted Mia-PaCa2 cells. Semiquantitative analysis revealed no significant difference between tumors overexpressing or not REG4, or treated with REG4 antibody, all of them showing very limited stroma (data not shown). However, our results could be explained, at least in part, by the fact that levels of the antiapoptotic-associated proteins such as activated AKT, Bcl-2, Bcl-xL and surviving, as well as the cell cycle-associated protein cyclin D1, were strongly decreased (Figure 7) indicating that apoptosis and cell cycle are regulated by reg4. Because antibodies are not supposed to penetrate tumor cells in large amounts, an autocrine or paracrine effect must be considered. Upon secretion by tumor cells, reg4 would activate, probably through specific receptors, intracellular pathways that favor cancer progression. These data are in agreement with results found in colon and gastric cancer cells *in vitro* [21], [22]. Altogether, these results suggest that overexpression of the REG4 protein, induced by its early gain in gene copy number, plays a major role in pancreatic tumor development and resistance to anticancer drugs. Targeting circulating REG4 protein appears as a promising adjuvant to current therapies of pancreatic adenocarcinoma, based on gemcitabine administration.

## Materials and Methods

### Study of the *reg4* gene copy number in pancreatic cancer

Fourteen consecutive pancreatic cancer samples were obtained by endoscopic ultrasound (EUS)-guided fine needle aspiration cytology between June 2007 and September 2007 at the Hospital Nord, Marseille. Written informed consent was obtained from all participants. Six samples were obtained from patients without detectable metastasis whereas 8 presented with metastases at the time of the punction. DNA was extracted, amplified and hybridized on Affymetrix Genome-Wide human SNP array 6.0 according to the manufacturer's instructions (Affymetrix Inc.). The Affymetrix Genome-Wide Human SNP Array 6.0 features more than 1.8 million markers of genetic variation, including more than 906,600 single nucleotide polymorphisms (SNPs) and more than 946,000 probes for the detection of copy number variation. The median inter-marker distance taken over all 1.8 million SNP and copy number markers combined is 696 bases. The array also contains 202,000 probes targeting 5,677 known regions of copy number variation, resolve into 3,182 distinct, non-overlapping segments, from the Toronto Database of Genomic Variants. Hybridization, washing, staining, and chip scanning were performed by the CRCHUL microarray Core Facility using materials and methods provided by the manufacturer (Affymetrix Inc.).

Overall hybridization quality was estimated by the call rate index obtained from GeneChip Genotyping Analysis Software (GTYPE, birdseed algorithm using default parameter settings). Allelic ratios were calculated with the Partek Genomics Suite, version 6.4 (Partek Inc., St. Louis, MO) using the proprietary default parameters. A 270 HapMap samples was used to create copy number from baseline. Genomic segmentation was utilized as a method to detect copy number alterations. Regions were detected using the following segmentation parameters: minimum of 10 genomic markers; segmentation p-value threshold lower than 0.001; and a signal to noise equal to 0.3. Using these parameters, 10263 segments were detected. Selected segments were visualized in a genomic context with the Partek® Genomics Suite.

### DNA from PanIN lesions

Seven micron sections from formalin-fixed, paraffin embedded tissue blocks were stained with hematoxylin and eosin. Homogeneous populations of cells were carefully microdissected using a PixCell II Laser Capture Microdissection System (Arcturus, Mountain View, CA) according to the manufacturer's instructions. PanINs were graded 1 to 3 according to current recommendations (http://pathology.jhu.edu/pancreas_panin/ and [29]) as a function of the importance of architectural and cytological abnormalities. Seven human pancreatic samples were analyzed in which we systematically found PanIN2 and PanIN3 lesions but PanIN1 lesions were found in only 6 of them. A total of >300 cells was collected for each category from serial tissue sections. Collected cells were transferred to an Eppendorf tube and resuspended in 20–50 µl of lysis buffer containing 10 mM Tris, 1 mM EDTA, 0.5% Tween 20 (pH 8.3), and 5 µl of proteinase K (20 mg/ml). Samples were incubated 24 hours at 55°C followed by boiling for 10 min to inactivate proteinase K. DNeasy Tissue Kit (Qiagen) was used to extract DNA according to the manufacturer's protocol. Extracted DNA was quantified using a NanoDrop ND-1000 spectrophotometer (Nano-Drop Technologies, Wilmington, DE).

### *reg4* gene copy number estimation by Real-Time PCR

*reg4* gene copy number was determined using the hreg4-F: 5′-TTTACTCCCTGTGGTCTGGG-3′ and hreg4-R: 5′-CTCTTTTCTCCAGCAAGGCA-3′ primers. Amplification was performed in a LigthCycler 480 (Roche Diagnostic) using the following schedule: 10 s denaturation at 95°C, 45 cycles of 8 s denaturation at 95°C, 7 s annealing at 60.5°C and 14 s of extension at 72°C. Melting curve was obtained by heating at 20°C/s to 95°C, cooling at 20°C/s to 65°C and heating at 0.1°C/s to 95°C with fluorescence data collection at 0.1°C intervals. Standard curves were generated using a 10-fold dilution series ranging from 0.1 ng to 100 ng. We performed qPCR for each individual in triplicate and determined the normalized relative copy number by generating a standard curve and normalizing across samples. PCR results were analyzed using the RealQuant software (Roche Diagnostic). The same 14 DNA samples from pancreatic cancer used for hybridization on the Affymetrix Genome-Wide human SNP array 6.0 were used for qPCR. DNA from blood leucocytes obtained from apparently healthy individuals was used as control.

### Retroviral vector and retroviral-mediated gene transfer

The entire coding sequence of reg4 cDNA was amplified by PCR using the primer pair 5′-GGGAATTCATGGCTTCCAGAAGCATGCGG-3′ (forward) and 5′-GGCTCGAGCTATGGTCGGTACTTGCACAGG-3′ (reverse), which contained EcoRI and XhoI restriction sites (underlined). The product was subcloned into EcoRI-XhoI restriction sites of the pLPCX retroviral vector obtained from S. Lowe (Cold Spring Harbor Laboratory, NY). Phoenix Amphotropic packaging cells (10^6^) were plated in a six-well plate, incubated for 24 h and transfected with polyethyleneimine with 5 µg of retroviral plasmid. Forty-eight hours later, the medium containing the virus was filtered (0.45 µm filter, Millipore) to obtain the first supernatant. Target Mia-PaCa2 were plated at 2×10^5^ cells per 35-mm dish and incubated overnight. For infection, the culture medium was replaced by an appropriate mix of the first supernatant and culture medium (v/v), supplemented with 4 g/ml polybrene (Sigma), and cells were incubated at 37°C. The population of reg4-expressing Mia-PaCa2 (Mia-PaCa2/reg4) was isolated by selection in the presence of puromycin (1 mg/ml). Mia-PaCa2 infected with the empty vector (Mia-PaCa2/empty) was used as control.

### *in vitro* studies

#### Cell culture conditions and siRNA transfection

The pancreatic cancer cell lines Mia-PaCa2/reg4 and Mia-PaCa2/empty were grown in Dulbecco's modified Eagle's medium (DMEM) supplemented with 10% fetal bovine serum and 2 mM L-glutamine in a humidified 5% CO_2_ atmosphere. The day before siRNA transfection, cells were plated in 6-well plates to eventually give 30–50% confluence. After removal of the medium, cells were washed once with serum-free medium and transfection was done in serum-free medium by addition of a mix composed of 10 µl Oligofectamine Reagent (Invitrogen) and 200 pmoles siRNA (reg4 siRNA [5′CTTCAGGAAGCTGAGGAAC3′] or control siRNA [5′AATTCTCCGAACGTGTCACGT3′] targeted sequences) diluted in 240 µl serum-free medium. After an incubation period of 4 hours at 37°C, the transfection medium containing siRNAs was replaced by fresh medium.

#### Cell growth and gemcitabine treatment

10^4^ cells/well were seeded on 96-well plates in 100 µl of culture medium. The next day, gemcitabine (purchased from Eli Lilly) was added in 100 µl of medium to a final concentration of 50 µM in the presence or not of 1 µg of reg4 monoclonal antibody. After 48 hours, 20 µl MTS ([3-(4,5-dimethylthiazol-2-yl)-5-(3-carboxymethoxyphenyl)-2-(4-sulfophenyl)-2H-tetrazolium] reagent obtained from Promega) was added, the plates were incubated at 37°C for 30 min, and the absorbance read at 490 nm.

### *in vivo* studies

#### Assessment of *in vivo* tumor growth

Institutional guidelines for the proper and human use in research were followed. Approximately 2×10^6^ Mia-PaCa-2 cells were inoculated subcutaneously with 0.1 ml of Matrigel (BD Biosciences Discovery Labware) to 6-week-old male nude mice. One day before tumoral cell inoculation, the control group received an intraperitoneal injection of 150 µl of PBS (vehicle), whereas the assay group received 0.25 mg of reg4 monoclonal antibody in 150 µl of PBS. Vehicle buffer or anti-reg4 antibody was injected weekly in animals. Tumor dimensions were measured once weekly and tumor volumes calculated with the formula length × width × depth ×0.5236 as previously reported [30]. Gemcitabine (100 mg/kg) was injected intraperitoneally twice a week. All studies were performed in accordance with the European Union regulations for animal experiments. The experiments were approved by the ethics committee at Marseille University.

#### Western blot analysis

Mia-PaCa2 cells and tumor tissue samples were lysed in a homogenization buffer containing pepstatin (1.45 mM), leupeptin (2.1 mM), DTT, triethanolamine (50 mM) and EDTA/EGTA (0.1 mM). Cell debris and insoluble materials were eliminated by centrifugation (14,000 g, 30 minutes at 4°C) and soluble fractions were either immediately assayed or stored at −80°C until use. Total protein (50 µg) was loaded in Laemmli buffer onto a 12.5% SDS-polyacrylamide gel in a Mini Cell (Bio-Rad). The proteins were transferred to nitrocellulose membranes for 1 hour using a Mini Trans-Blot Electrophoretic Transfer Cell (Bio-Rad). The membrane was blocked in 1x PBS/0.05% Tween 20/5% nonfat dry milk overnight at 4°C. After two washes in 1x PBS/0.005% Tween 20, primary antibodies were added for 1–2 hours. After three more washes, the secondary antibody was added for 1.5 hour. The membrane was developed using the enhanced chemiluminescence (ECL) kit (Amersham Life Science) on Kodak film in the dark room. The membrane was then stripped and reprobed for β-actin by using the protocol described in the ECL kit.

#### Antibodies

Anti-human reg4 monoclonal antibody (Clone 200214) was purchased from R&D Systems; Bcl-2 (C21), Bcl-xL (H-62), survivin (FL-142), cyclin D1 (C20) and β-actin (N-21) rabbit polyclonal antibodies were from Santa Cruz Biotechnology; phosphorylated AKT (phospho-Ser473) polyclonal antibody was from Upstate Biotechnology Inc; pan-AKT monoclonal antibody (clone 40D4) was from Cell Signaling Technology.

#### Statistical analysis

All results were expressed as mean +/− SE. Statistical analysis was performed by a one way ANOVA followed by Fisher's protected least significant difference (PLSD) test (Statview 512, Brain Power Inc., Calabases, CA). Values of p<0.05 were considered statistically significant.
